# Relationship between fibrosis induced by preoperative chemoradiotherapy and real-time tissue elastography of internal anal sphincter

**DOI:** 10.1007/s00384-025-05026-1

**Published:** 2025-11-08

**Authors:** Akira Sakamoto, Kazuhito Sasaki, Hiroyuki Abe, Ryosuke Kikuchi, Hiroaki Nozawa, Koji Murono, Shigenobu Emoto, Yuichiro Yokoyama, Yuzo Nagai, Shinya Abe, Takahide Shinagawa, Yuichi Tachikawa, Satoshi Okada, Tetsuo Ushiku, Soichiro Ishihara

**Affiliations:** 1 Department of Surgical Oncology, Faculty of Medicine, The University of Tokyo, Tokyo, Japan; 2 Department of Pathology, Faculty of Medicine, The University of Tokyo, Tokyo, Japan

**Keywords:** Colorectal cancer, Preoperative chemoradiotherapy, Real-time tissue elastography

## Abstract

**Background:**

It has been suggested that chemoradiotherapy may cause fibrosis of the internal anal sphincter, resulting in sclerosis. However, no report has quantitatively investigated this relationship by using real-time tissue elastography.

**Objective:**

To clarify the relationship between fibrosis and elasticity of the internal anal sphincter in patients undergoing surgery for lower rectal cancer with or without preoperative chemoradiotherapy from a histological perspective.

**Design:**

A single-center, prospective cohort study.

**Settings:**

The surgical and pathological departments in a tertiary referral university hospital.

**Patients:**

Eighteen patients with rectal cancer who underwent abdominoperineal resection between May 2019 and May 2022 were included in the study.

**Main outcome measures:**

Real-time tissue elastography was performed before surgery to measure internal anal sphincter hardness as “elasticity” (hardest (0) to softest (255); decreased elasticity indicated sclerotic changes). Internal anal sphincter fibrosis was evaluated using Masson’s trichrome and Elastica van Gieson staining. We evaluated internal anal sphincter elasticity after preoperative chemoradiotherapy and preoperatively in patients who did not undergo preoperative chemoradiotherapy and analyzed the correlation with the percentage of internal anal sphincter fibrosis in the resected specimens.

**Results:**

Of the 18 patients, 10 underwent preoperative chemoradiotherapy. A significantly higher percentage of internal anal sphincter fibrosis was observed in the chemoradiotherapy group. Post-chemoradiotherapy elasticity was significantly lower in patients undergoing chemoradiotherapy compared to pre-chemoradiotherapy elasticity and that in patients not undergoing chemoradiotherapy. The analysis of the correlation between internal anal sphincter elasticity and fibrosis showed that elasticity decreased as the percentage of fibrosis increased.

**Limitations:**

This study was conducted at a single institution, and the number of cases was small. The radiation dose to the anal canal may have differed depending on the location of the tumor, which may have affected internal anal sphincter elasticity.

**Conclusions:**

Internal anal sphincter elasticity may reflect tissue sclerosis associated with fibrosis caused by chemoradiotherapy.

**Supplementary Information:**

The online version contains supplementary material available at 10.1007/s00384-025-05026-1.

## Introduction

Colorectal cancer is the second leading cause of cancer-related deaths worldwide, accounting for 10% of all cancer types [1]. Preoperative chemoradiotherapy (CRT) followed by total mesorectal excision and postoperative adjuvant chemotherapy has become the standard of care for patients with locally advanced rectal cancer [2, 3]. Furthermore, total neoadjuvant therapy, a combination of radiation therapy and chemotherapy, is now recommended as an option in the National Comprehensive Cancer Network (NCCN) guidelines [4, 5].

While some reports have indicated that preoperative CRT has an impact on the development of anorectal dysfunction and fecal incontinence after rectal surgery, others have indicated that preoperative CRT has no significant impact on defecation, an issue that is still controversial [6–11].

Previously, we established a method to quantify the hardness of the internal anal sphincter (IAS) as elasticity using real-time tissue elastography (RTE) on endoanal ultrasound (EAUS) [12]. This method has made it possible to objectively quantify the sclerosis of the IAS, especially before and after CRT. By using RTE on IAS, we reported that IAS sclerosis with preoperative CRT was associated with increased maximum rest pressure and worsened Cleveland Clinic Florida Fecal Incontinence Score (CCFIS) [13]. Since tissue sclerosis generally correlates with fibrosis [14, 15], we hypothesized that IAS sclerosis measured by RTE might also be related to IAS fibrosis induced by CRT. In this study, we aimed to clarify the relationship between IAS fibrosis and elasticity as an indicator of IAS sclerosis in patients with lower rectal cancer who underwent abdominoperineal resection (APR) from a histological aspect.

## Materials and methods

### Patients

We consecutively enrolled 18 patients with locally advanced rectal cancer whose IAS elasticity was measured preoperatively using EAUS and who underwent APR between May 2019 and May 2022 at the Department of Surgical Oncology, Graduate School of Medicine, The University of Tokyo. Data regarding patient background, including age, sex, tumor location from the anal verge before CRT, histology, history of anal surgery, clinical T Stage, and clinical N stage, were collected from medical records. For patients who underwent preoperative CRT, elasticity was measured before and after preoperative CRT. Of the 18 patients, 10 received preoperative CRT.

### Ethics approval

The study protocol was approved by The University of Tokyo Ethics Committee (No. 10046-(5) and No. 3252-(16)). Written informed consent was obtained from all patients.

### Preoperative chemoradiotherapy

The indication for CRT was primary adenocarcinoma of the lower rectum (cT3-cT4, any N, M0) without distant metastasis and located below the peritoneal reflection (second Houston valve). The staging was based on the American Joint Committee on Cancer staging system, 8th edition [16]. CRT consisted of a total dose of 50.4 Gy of radiation using the 4-field box technique and concomitant 5-fluorouracil-based chemotherapy. Surgery was performed 8–10 weeks after the completion of CRT. All patients underwent APR for radical surgery based on total mesorectal excision.

### Histological staining and image acquisition

To assess IAS fibrosis, tissue including the IAS in rectal resection specimens was fixed, embedded in paraffin, cut into 5-μm slices, and stained with Masson’s trichrome (MT) and Elastica van Gieson (EVG) stains. In 16 cases, tissue was obtained from the tumor contralateral side, and in two cases, tissue was obtained from the non-contralateral side.

**Fig. 1:**
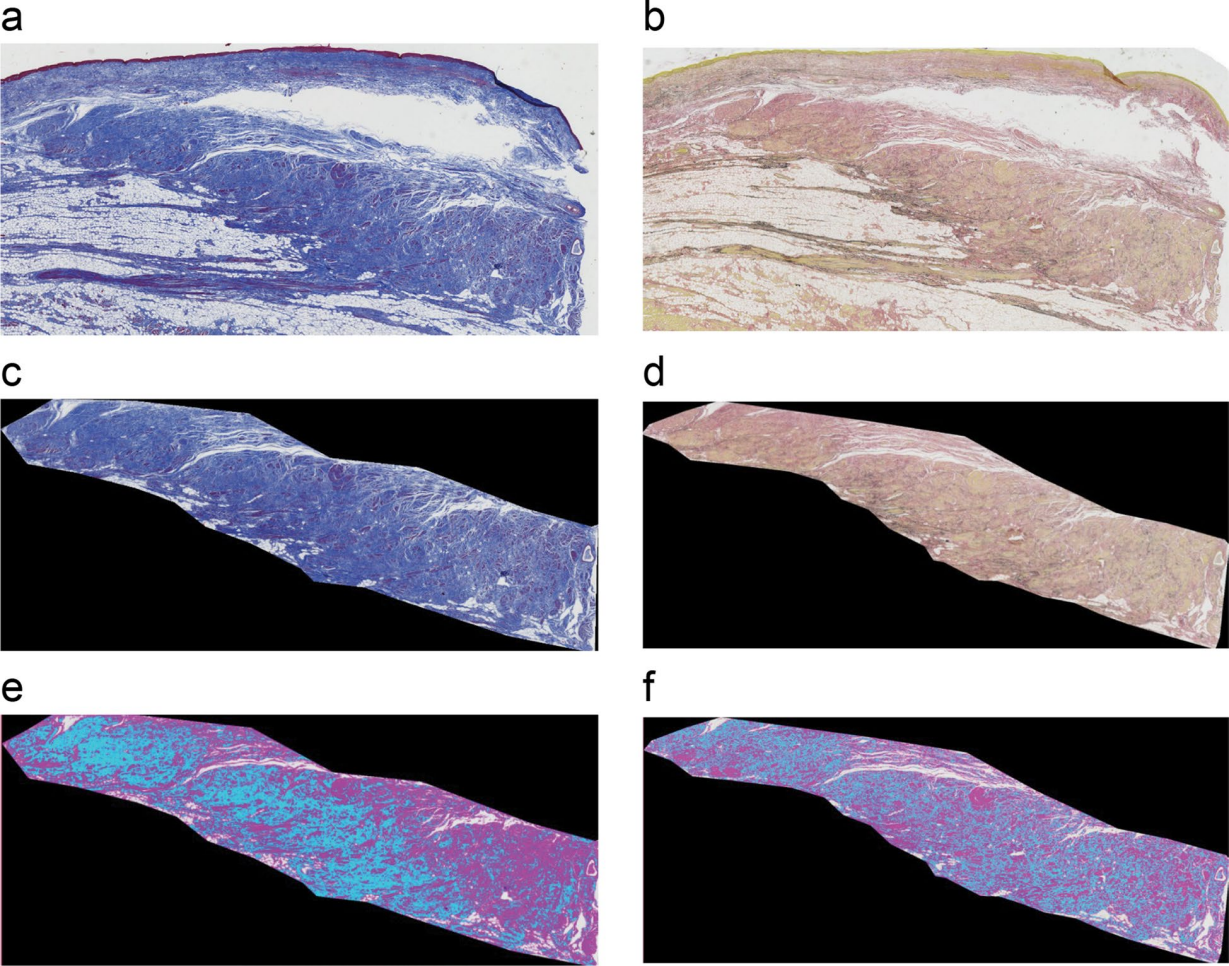
Calculation of fibrosis percentage. **a** Masson’s trichrome (MT) and **b** Elastica van Gieson (EVG) staining of the internal anal sphincter (IAS) in the rectal specimen. **c** Extraction of IAS areas for MT staining and **d** EVG staining. **e** The blue areas indicate the collagen fibers for MT staining and **f** EVG staining

The degree of fibrosis was observed in both groups, and all images of the MT- and EVG-stained sections were captured with an all-in-one fluorescence microscope (BZ-X800, KEYENCE, Osaka, Japan) using a Plan Apochromat 2 × objective (NA0.75, BZ-PA02, KEYENCE, Osaka, Japan). The percentage of stained collagen fibers relative to the total IAS area in the sections was defined and calculated as the percentage of fibrosis. Figure 1 presents MT- and EVG-stained images of the IAS and images analyzed to calculate the area of fibrosis. The percentage of fibrosis was calculated by extracting the range of collagen fibers stained blue by MT staining and the range stained reddish brown by EVG staining.

### Real-time tissue elastography

**Fig. 2:**
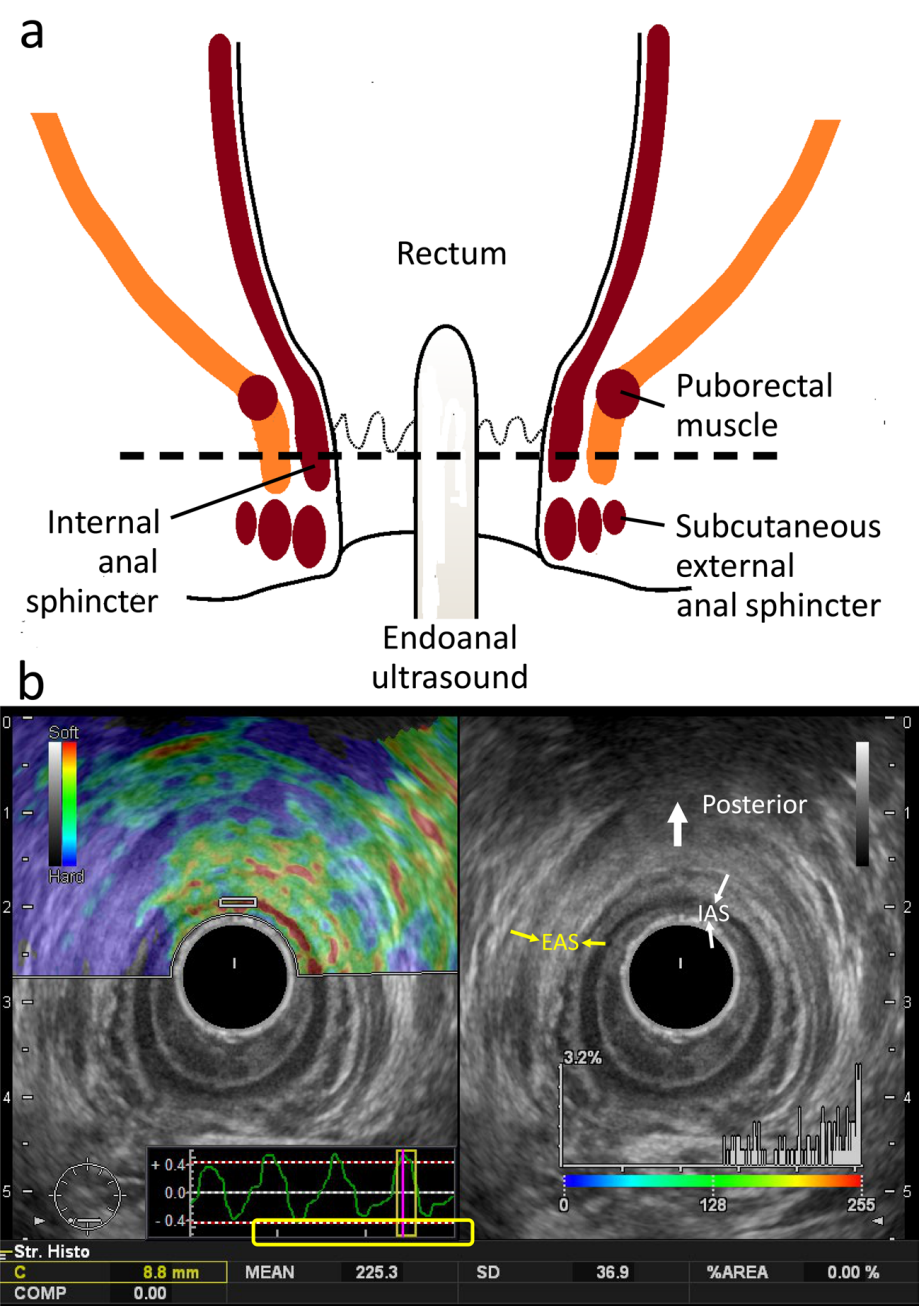
Internal anal sphincter evaluation. **a** Diagram showing the anal canal and elastography measurement site. **b** Endoanal ultrasonography demonstrating the internal (IAS; white arrows) and external (EAS; yellow dotted arrows) anal sphincters

B-mode ultrasound imaging and RTE measurements of the anal canal were performed using a Noblus ultrasound system (Hitachi, Tokyo, Japan) equipped with a rectal probe (EUP-R54AW-19) and 10-MHz transducer. All ultrasound examinations, including RTE, were performed by two different surgeons. All patients were placed in the left lateral recumbent position and a rectal probe was inserted into the anal canal for measurement (Fig. 2a). RTE at the IAS of each patient was performed with freehand manual compression in the anterior, left, posterior, and right positions to determine whether the IAS was well delineated. If IAS elasticity could be assessed, the RTE technique was performed according to the procedure previously reported by Fukui et al. [12] Real-time tissue elastography was performed at the level of the anal canal between the puborectalis muscle and the subcutaneous external anal sphincter. IAS elasticity at the contralateral side of the tumor was recorded and evaluated. Elasticity was expressed as a color scale from 0 to 255 and numerical value, with lower values indicating stiffer tissue and higher values indicating softer tissue. We identified the region of interest in the IAS and calculated the mean value of the strain histogram, which we defined as elasticity. Representative images of IAS evaluation in the posterior position are shown in Fig. 2b. Pre- and post-CRT IAS elasticity were recorded 1–3 weeks before initiation and 4–6 weeks after the end of CRT, respectively (Supplemental Fig. 1). For the group that did not receive CRT, preoperative elasticity was measured and included in the study. The correlation between IAS fibrosis percentage in the resected specimens and RTE of the IAS was examined. We also compared IAS elasticity and fibrosis rates between the two groups based on whether preoperative CRT was performed or not.

### Statistical analysis

Quantitative data are expressed as means ± standard deviations, and scores are expressed as medians and ranges. Comparisons were made using Fisher’s exact test for categorical variables and the Mann–Whitney *U* test or Wilcoxon signed-rank test for continuous variables. Statistical correlations for IAS elasticity and fibrosis percentage were made using Spearman’s rank correlation coefficient. All analyses were performed using JMP Pro 15.0.0 software (SAS Institute Inc., Cary, NC, USA), and *p* < 0.05 was considered significant for all analyses.

## Results

A comparison of the clinicopathological factors between the two groups classified according to the presence or absence of preoperative CRT is shown in Table 1. Ten patients were in the CRT group and eight were in the non-CRT group. There were no significant differences between groups in terms of age, sex, body mass index, distance from the tumor to the anal verge, or clinical stage. In both groups, no patient had a history of anorectal surgery.

**Table 1:** Patient characteristics

Clinicopathological characteristic	Variable	CRT (+) (*n* = 10)	CRT (−) (*n* = 8)	*p*
Age (years)	Median (range)	68 (51–84)	64 (44–97)	0.29
Sex	Male/female	7/3	5/3	1.00
BMI, kg/m^2^	Median (range)	21.6 (16.7–28.5)	23.4 (17.2–25.9)	0.39
Previous anorectal surgery	Yes/no	0/10	0/8	N/A
Tumor location from AV (cm)	Median (range)	2 (1–4)	2 (1–3)	0.92
Histology	Well to moderately differentiated/others	9/1	6/2	0.56
Clinical T-stage	cT0-2/cT3-4	0/10	3/5	0.07
Clinical N-stage	cN0/cN1-2	5/5	6/2	0.37

Continuous variables are expressed as medians (ranges)

*CRT* chemoradiotherapy, *BMI* body mass index, *AV* anal verge

**Fig. 3:**
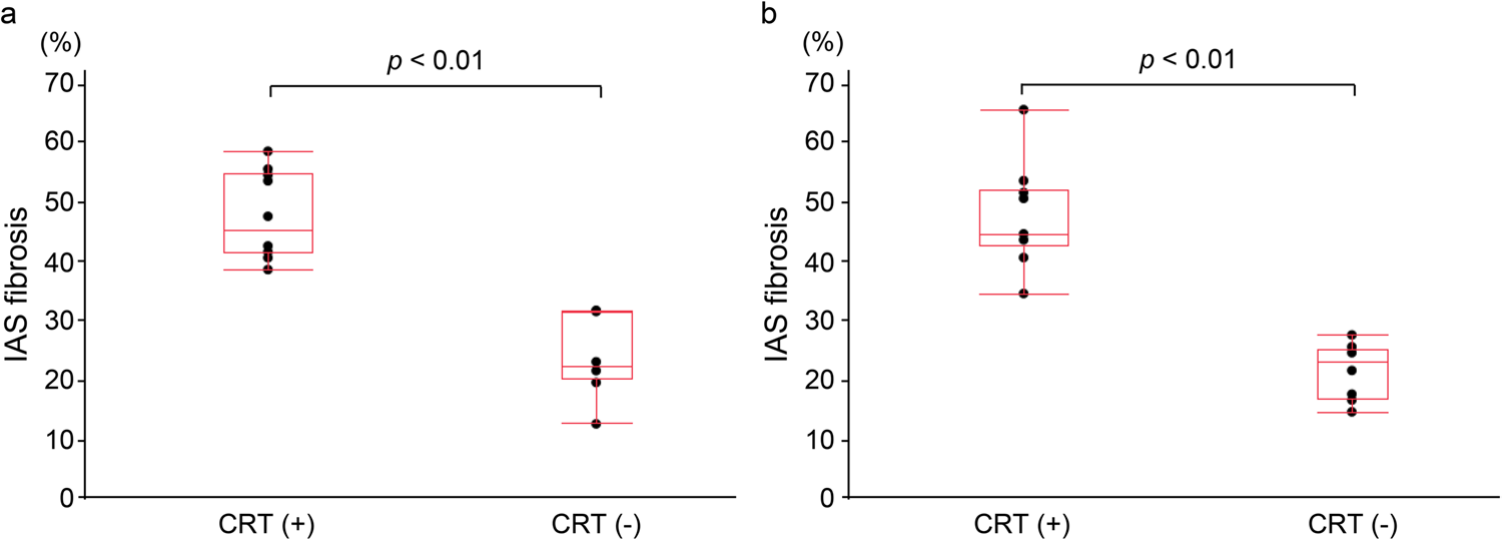
Comparison of the internal anal sphincter fibrosis percentage between CRT and non-CRT groups using **a** Masson’s trichrome and **b** Elastica van Gieson staining

Regarding IAS fibrosis assessed using both MT and EVG staining, a significantly higher fibrosis was observed in the CRT group than in the non-CRT group (MT staining, median: 46% vs. 23%, *p* < 0.01; EVG staining: 45% vs. 24%, *p* < 0.01; Fig. 3a, b).

**Fig. 4:**
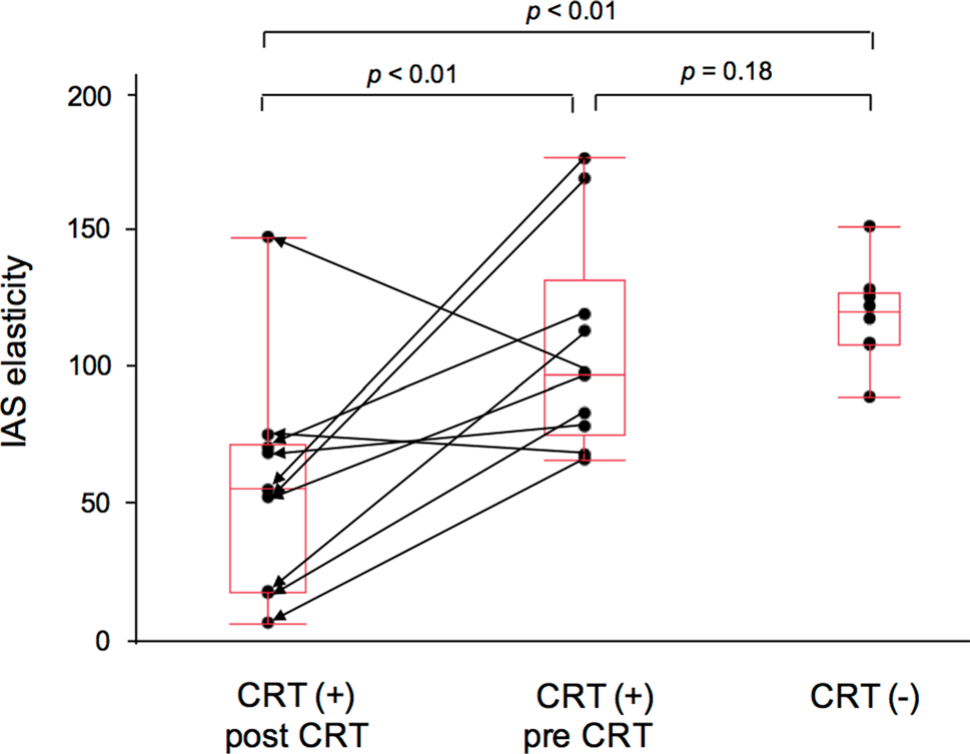
Analysis of internal anal sphincter elasticity between the chemoradiotherapy (CRT; before and after CRT) and non-CRT groups

Comparing pre- and post-CRT elasticity in the CRT group and preoperative elasticity in the non-CRT group, post-CRT elasticity in the CRT group was significantly lower than pre-CRT (median: 54.4 vs. 96.5, *p* < 0.01), which was also lower than that in the patients who did not undergo CRT (median: 54.4 vs. 119.2, *p* < 0.01; Fig. 4). There was no significant difference between the pre-CRT elasticity of the CRT group and preoperative elasticity of the non-CRT group (96.5 vs. 119.2, *p* = 0.18).

**Fig. 5:**
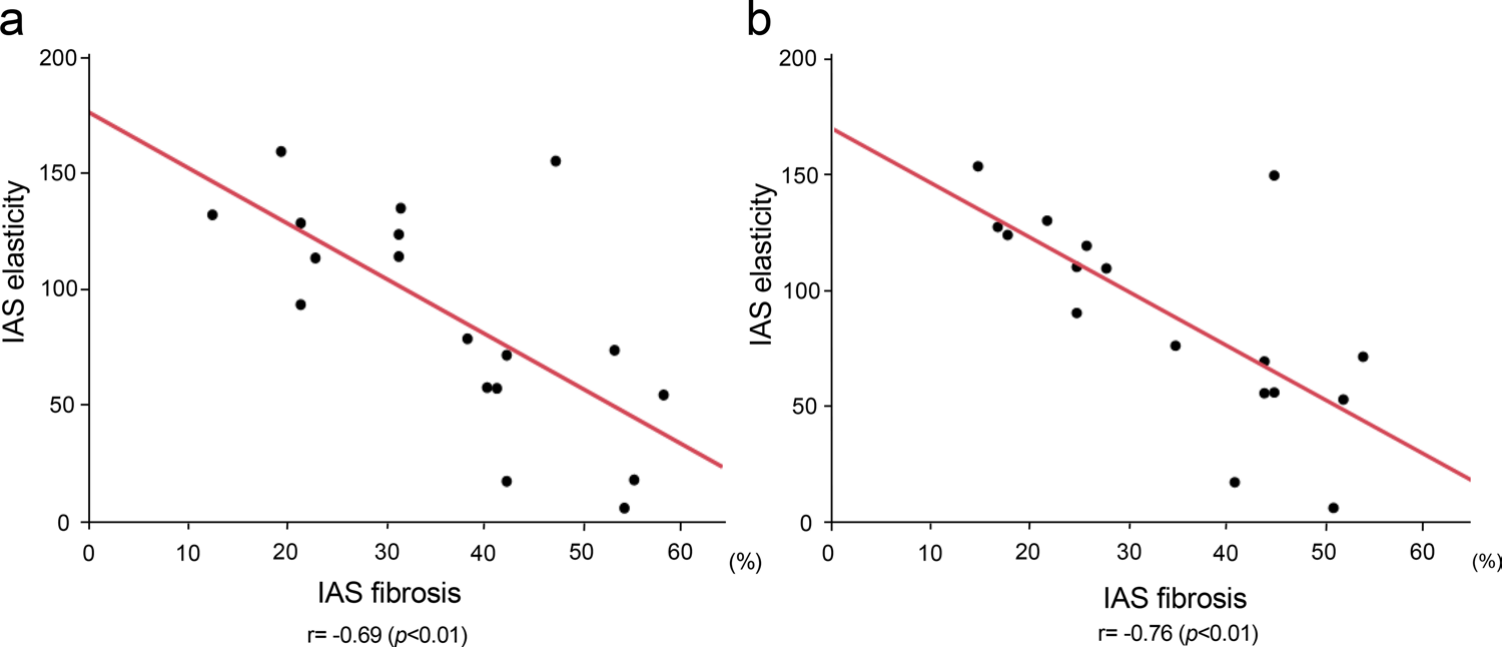
Correlation between internal anal sphincter (IAS) elasticity and fibrosis area percentage of the IAS measured using **a** Masson’s trichrome and **b** Elastica van Gieson staining

Notably, the inverse correlation between IAS elasticity and fibrosis was confirmed in all preoperative cases, which showed that the percentage of fibrosis in IAS increased as the tissue of IAS became more sclerotic. This trend was confirmed by MT and EVG staining (MT, *r* = −0.69, *p* < 0.01; EVG, *r* = −0.76, *p* < 0.01; Fig. 5a, b).

## Discussion

In the present study, the CRT group showed a significantly higher percentage of fibrosis and significantly lower IAS elasticity than the non-CRT group. Additionally, a decrease in IAS elasticity was strongly correlated with tissue fibrosis. RTE was originally developed and clinically applied to diagnose breast cancer based on tissue hardness, but has also been applied to diagnose the extent of liver cirrhosis because progressive fibrosis is a pathological feature of normal tissue exposed to radiation [17–19]. However, no other previous studies have focused on the RTE of IAS except for our institution, and to the best of our knowledge, this is the first study to quantify IAS sclerosis using RTE and to confirm its strong correlation with IAS fibrosis.

In general, adverse events from radiation therapy occur at the start of irradiation and increase in a time-dependent manner, and RT-induced fibrosis is a well-known side effect that can arise in some patients [20, 21]. In a previous report on the histological changes caused by preoperative CRT in the IAS, Da Silva et al. showed that fibrosis of the IAS was significantly increased in patients who received preoperative CRT compared to those who did not. [11] They further reported that this histological change in the sphincter muscle may affect the stiffness and tone of the IAS, which may cause increased fecal incontinence [11]. Koushi et al. conducted a retrospective study on 95patients with lower rectal cancer who underwent preoperative CRT, neoadjuvant chemotherapy (NAC), or surgery alone. This previous report also concluded that peritoneal fibrosis was more pronounced in the preoperative CRT group than in the NAC or surgery alone groups [22]. We examined the effect of CRT on the IAS by using two different stains to assess the percentage of IAS fibrosis, mainly on the contralateral side of the tumor, and also found increased IAS fibrosis.

The results of our study indicate a decrease in elasticity, also known as tissue sclerosis, in patients who received preoperative CRT, which is a new finding. The strong correlation between increased fibrosis and IAS sclerosis shown in this study indicates that a decrease in IAS elasticity may suggest an increase in local fibrosis due to preoperative CRT. We have previously demonstrated that decreased elasticity of the IAS following CRT leads to worse Cleveland Clinic Florida Incontinence Scores [13]. The present study provides the first evidence that anorectal dysfunction after CRT may be attributable to radiation-induced fibrosis and sclerotic changes in the IAS.

Radiotherapy provides significant benefits but can lead to irreversible side effects such as radiation-induced fibrosis, for which no curative treatment exists; management remains symptomatic [23, 24]. While radiation dose and exposed tissue volume are key risk factors, genetic predisposition also plays a role [25, 26]. The impact of dose variation due to tumor location and individual sensitivity on IAS elasticity and fibrosis requires further study.

This study has several limitations. First, it was conducted at a single institution and the number of cases was small. Although reproducibility was examined in the previous study [12], elasticity measurements were conducted by only two physicians, which may have influenced the results. Second, this study focused only on collagen fibers. Fibrosis can be evaluated using indicators such as fibroblast and myofibroblast activity (e.g., α-smooth muscle actin [α-SMA]), extracellular matrix proteins (e.g., fibronectin, elastin, and laminin), and molecular markers including transforming growth factor-β (TGF-β). Therefore, further studies incorporating these markers will likely be needed for a more comprehensive evaluation. Third, since excised specimens were used for analysis, the timing of IAS elasticity measurement and the rate of fibrosis in the IAS might have been different, potentially introducing error in the relationship between the two.

In conclusion, IAS elasticity may reflect tissue stiffness associated with fibrosis caused by CRT.

## Supplementary Information

Below is the link to the electronic supplementary material.

Supplemental Fig. 1 Timeline of IAS elasticity and fibrosis measurements in CRT and non-CRT groups. (PNG 182 KB)

## Data Availability

The data that support the findings of this study are not openly available due to reasons of sensitivity and are available from the corresponding author upon reasonable request.

## Declarations

### Paper category

Colorectal/anal neoplasia.
