# Supplementary figures and images for: Relationship between fibrosis induced by preoperative chemoradiotherapy and real-time tissue elastography of internal anal sphincter
Source: Int J Colorectal Dis. 2025 Nov 8;40(1):227. doi: 10.1007/s00384-025-05026-1 (PMC12594675; doi:10.1007/s00384-025-05026-1)

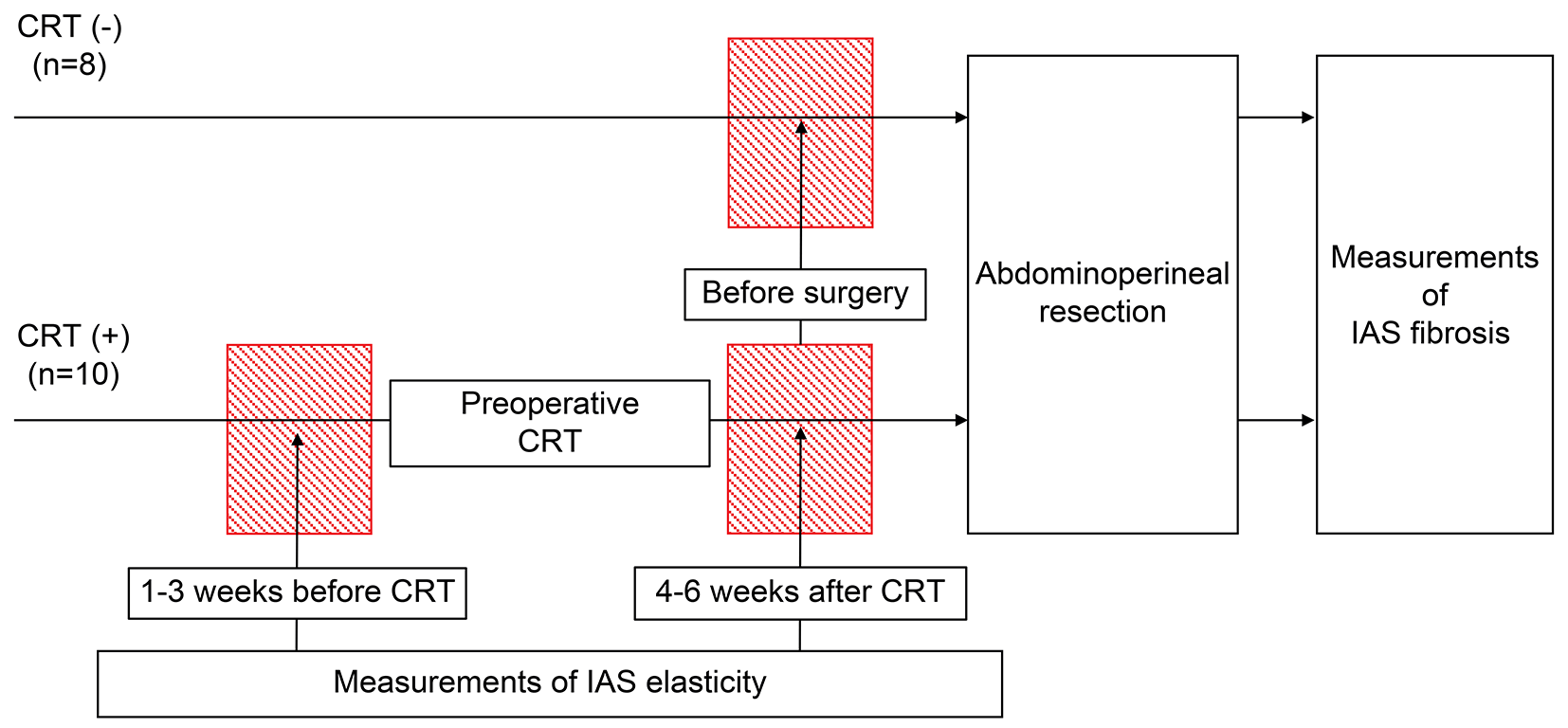

Supplement: Supplementary file 1 — Timeline of IAS elasticity and fibrosis measurements in CRT and non-CRT groups. (PNG 182 KB) [file 384_2025_5026_MOESM1_ESM.png]
